# Huntington's disease clinical trials update: March 2025

**DOI:** 10.1177/18796397251337000

**Published:** 2025-04-30

**Authors:** Mena Farag, Sarah J Tabrizi, Edward J Wild

**Affiliations:** Huntington's Disease Centre, UCL Queen Square Institute of Neurology, London, UK

**Keywords:** Huntington's disease, clinical trials

## Abstract

In this edition of the Huntington's Disease Clinical Trials Update, we expand on the ongoing phase I clinical trial of ALN-HTT02 from Alynlam Pharmaceuticals. We also report on the SAGE-718 (also known as dalzanemdor) program from Sage Therapeutics, with results of the phase II DIMENSION study and the recent termination of the open-label phase III PURVIEW study. Additionally, we discuss recent developments in the regulatory pathway for AMT-130, following discussions between uniQure and the U.S. Food and Drug Administration regarding key aspects of accelerated approval. Finally, we provide a comprehensive listing of all currently registered and ongoing clinical trials in Huntington's disease.

## Plain language summary

In this edition we expand on the ongoing phase I clinical trial of ALN-HTT02 from Alynlam Pharmaceuticals. We also report on the SAGE-718 (also known as dalzanemdor) program from Sage Therapeutics, with results of the phase II DIMENSION study and the recent termination of the open-label phase III PURVIEW study. Additionally, we discuss recent developments in the regulatory pathway for AMT-130, following discussions between uniQure and the U.S. Food and Drug Administration regarding key aspects of accelerated approval. Finally, we provide a comprehensive listing of all currently registered and ongoing clinical trials in Huntington's disease.

## Introduction

The Clinical Trials Update is a regular feature devoted to highlighting ongoing and recently completed clinical trials in Huntington's disease (HD). Clinical trials previously reviewed in this section are listed in Table 1.

**Table 1.** Clinical trials previously reviewed by the Huntington's Disease Clinical Trials Update.

	Trial Name	Intervention	Edition
NCT02519036	IONIS-HTTRx	IONIS-HTT_Rx_^a^	September 2017^1^
NCT02215616	LEGATO-HD	Laquinimod	
NCT02197130	Amaryllis	PF-02545920	
NCT02006472	PRIDE-HD	Pridopidine	
NCT03225833	PRECISION-HD1	WVE-120101	February 2018^2^
NCT03225846	PRECISION-HD2	WVE-120102	
NCT01795859	FIRST-HD	Deutetrabenazine	
NCT02481674	SIGNAL	VX15/2503	August 2018^3^
NCT00712426	CREST-E	Creatine	
NCT03761849	GENERATION-HD1	RG6042^a^	January 2019^4^
NCT03344601	PACE-HD	Physical activity	
NCT02535884	HD-DBS	Deep brain stimulation	June 2019^5^
NCT02453061	TRIHEP3	Triheptanoin	
NCT04120493	AMT-130	AAV5-miHTT	April 2020^6^
NCT04102579	KINECT-HD	Valbenazine	
NCT05111249	VIBRANT-HD	Branaplam	April 2022^7^
NCT04514367	ANX005	ANX-005	
NCT04514367	SHIELD HD	Observational study	
NCT03761849	GENERATION-HD1	Tominersen^a^	
NCT05032196	SELECT-HD	WVE-003	
NCT03225833	PRECISION-HD1	WVE-120101	
NCT03225846	PRECISION-HD2	WVE-120102	
NCT02481674	SIGNAL	Pepinemab^b^	November 2022^8^
NCT05358717	PIVOT HD	PTC518	
NCT05686551	GENERATION HD2	Tominersen^a^	August 2023^9^
NCT05541627	AB-1001	AAVrh10.CAG.hCYP46A1^c^	
NCT05822908	VO659-CT01	VO659	March 2024^10^
NCT05111249	VIBRANT-HD	Branaplam	
NCT06254482	PIVOT HD	PTC518 (extension study)	September 2024^11^
* NCT06585449	**ALN-HTT02**	**ALN-HTT02**	**March 2025**
* NCT05107128	**DIMENSION**	**SAGE-718**	
* NCT05655520	**PURVIEW**		

^a^ IONIS-HTT_Rx_, RG6042 and tominersen refer to the same molecule.

^b^ VX15/2503 and pepinemab refer to the same molecule.

^c^ AAVrh10.CAG.hCYP46A1, BV-101 and AB-1001 refer to the same molecule.

* New clinical trials reviewed in the Huntington's Disease Clinical Trials Update.

In this edition, we expand on the phase I clinical trial of ALN-HTT02 (NCT06585449)^12^ from Alynlam Pharmaceuticals. We also report on the program studying SAGE-718 (also known as dalzanemdor) from Sage Therapeutics, with results of the phase II DIMENSION study (NCT05107128)^13^ and the recent termination of the open-label phase III PURVIEW study (NCT05655520).^14^ Additionally, we discuss recent developments in the regulatory pathway for the huntingtin-lowering gene therapy AMT-130, following discussions between its sponsor uniQure and the U.S. Food and Drug Administration regarding key aspects of accelerated approval.^15^ Finally, we provide a comprehensive listing of all currently registered and ongoing clinical trials in Huntington's disease.

We tabulate all currently registered and ongoing clinical trials in Tables 2–4. For further details on the methodology used, please refer to the first edition of this series.^1^

**Table 2.** Pharmacological clinical trials registered at the World Health Organization (WHO) International Clinical Trials Research Platform (ICTRP) for people with HD since the first edition of the “Clinical Trials Corner”.

Registration ID	Trial Name	Intervention	Mechanism of Action	Population	Comparison	Main Outcome	Study Design	Estimated Enrolment	Sponsor	Location(s)
NCT06585449*	-	ALN-HTT02	RNA interference selectively targeting exon 1 of *HTT* mRNA	HD-ISS stage 2 or early stage 3 HD	Placebo	Frequency of TEAEs in both the double-blind and open-label phases, monitored over 12 months	Randomized, double-blind, parallel assignment, single dose	54	Alnylam Pharmaceuticals	Canada, UK – will expand (multi-centre)
CTIS2024-514328-18-00*	VO659-CT01	Intrathecal administration of VO659	ASO targeting mutant *HTT* and ataxin RNA to reduce toxic protein levels	SCA1, SCA3, HD	None	Safety, tolerability, and pharmacokinetics of multiple doses	Open-label, non-randomized	23	Vico Therapeutics B.V.	Denmark, France, Germany, Israel, Netherlands, UK
CTIS2024-518875-73-00*	TEMET-HD	Metformin	Modulation of cellular energy metabolism via AMP-activated protein kinase activation to slow cognitive decline	HD	Placebo	Change in UHDRS cognitive score after 6 and 12 months	Randomized, double-blind, placebo-controlled	60	IIS La Fe	Spain (pending)
NCT06474650	-	LPM3770164	VMAT2 inhibitor	Healthy controls	None	Pharmacokinetics pre-dose and up to 240 h post-dose	Randomized, open-label, two-period, double-crossover (phase I) study	16	Luye Pharma Group Ltd	China (single centre)
NCT06469853	-	MBF-015	Histone deacetylase 1/2 inhibitor	Early and moderate HD	None	Safety and tolerability at 43 days	Open-label, single centre (phase IIa) study	10	Medibiofarma S.L.	Spain (single centre)
NCT06312189	-	Valbenazine	VMAT2 inhibitor	HD with chorea; participated in study NBI-98854-HD3006 (NCT04400331)	None	Number of participants with TEAEs up to week 106	Non-randomized, open-label	7	Neurocrine Biosciences	Canada (multi-centre)
NCT06254482	-	PTC518	Small molecule splicing modulator	Participants who completed the treatment period in PTC518-CNS-002-HD	None	Number of participants with TEAEs up to month 30; blood total HTT levels up to month 28	Randomized, double-blind, parallel assignment, extension (phase IIb) study	250	PTC Therapeutics	Australia, Austria, Canada, France, Germany, Italy, Netherlands, New Zealand, Spain, UK (multi-centre)
NCT06097780	-	Nestacell	Dental pulp stem cell	Early and moderate HD	Placebo	Efficacy at 1 year	Randomized, double-blind, parallel assignment, multiple dose	120	Azidus Brasil	N/S
NCT06024265	-	ER2001	Small interfering RNA	Early HD	None	Safety at 6.5 months	Multiple dose, open-label trial	15	ExoRNA Bioscience	China (single centre)
2022-001565-12	-	PTC518	Small molecule splicing modulator	Prodromal and early HD	None	Safety at 24 months, blood total HTT levels at 24 months	Randomized, double-blind, parallel assignment, multiple dose	250	PTC Therapeutics	France, Germany, Netherlands, UK, USA (multi-centre)
NCT05822908	-	VO659	CAG-targeting ASO	Early HD, mild-moderate SCA1, mild-moderate SCA3	None	Safety at 253 days	Open-label, non-randomized, sequential assignment, multiple ascending dose	65 (19 HD, 19 SCA1 and 27 SCA3)	VICO Therapeutics B.V.	France, Germany, Italy, Poland, the Netherlands, UK (multi-centre)
NCT04556656†	PROOF-HD	Pridopidine	Sigma-1 receptor activation	Early HD	Placebo	Change in function at 65 weeks	Randomized, double-blind, parallel assignment, single dose trial	499	Prilenia Therapeutics	Austria, Canada, Czechia, France, Germany, Italy, Netherlands, Poland, Spain, UK, USA (multi-centre)
NCT05686551	GENERATION HD2	Tominersen	Non-allele-selective ASO	Prodromal and early HD	Placebo	Safety at 24 months	Randomized, double-blind, dose-finding trial	360	Hoffmann-La Roche	USA, Spain, more sites to be confirmed (multi-centre)
NCT05655520†	PURVIEW	SAGE-718	Positive allosteric modulator of NMDA	Premanifest, early and moderate HD	None	Safety at 13 months	Single-dose open-label trial	153	Sage Therapeutics	Canada, USA (multi-centre)
NCT03019289†	-	Pridopidine	Sigma-1 receptor activation	Healthy controls, early and moderate HD	None	Sigma-1 receptor occupancy	Multiple dose, open-label trial	23	Prilenia Therapeutics / Teva	Germany (single centre)
NCT02494778†	Open PRIDE HD	Pridopidine	Sigma-1 receptor activation	Early and moderate HD	Placebo	Efficacy at 106 weeks	Open-label extension	400	Prilenia Therapeutics / Teva	Australia, Austria, Canada, France, Germany, Italy, Netherlands, Poland, Russia, UK, USA (multi-centre)
NCT02006472†	PRIDE HD	Pridopidine	Sigma-1 receptor activation	Early and moderate HD	Placebo	Efficacy at 26 weeks	Randomized, double-blind, parallel assignment, dose-finding trial	408	Prilenia Therapeutics / Teva	Australia, Austria, Canada, Denmark, France, Germany, Italy, Poland, Russia, Netherlands, UK, USA (multi-centre)
NCT01306929†	OPEN-HART	Pridopidine	Sigma-1 receptor activation	HD	None	Safety up to 72 months	Randomized, placebo-controlled, dose-ranging, parallel-group study.	134	Prilenia Therapeutics / Teva	Canada, USA (multi-centre)
NCT05509153	NAC-preHD	NAC	Antioxidant	Premanifest HD	Placebo	Efficacy at 36 months	Randomized, double-blind trial	160	Western Sydney Local Health District	Australia (multi-centre)
ISRCTN56240656†	FELL-HD	Felodipine	Calcium channel blocker	Early HD	None	Safety at 62 weeks	Non-randomised, multiple dose trial	18	Cambridge University	UK (single centre)
NCT05358821†	SURVEYOR	SAGE-718	Positive allosteric modulator of NMDA	Early and moderate HD	Placebo	Change in cognition at 28 days	Double-blind, placebo-controlled, single dose design trial	69	Sage Therapeutics	USA (multi-centre)
NCT05358717	PIVOT HD	PTC518	Small molecule splicing modulator	Prodromal and early HD	Placebo	Safety at 113 days	Randomized, double-blind, placebo controlled, parallel assignment, multiple dose trial	162	PTC Therapeutics	France, Germany, Netherlands, UK, USA (multi-centre)
NCT05475483	-	SOM-3355 (bevantolol hydrochloride)	Beta-blocker	Early and moderate HD	Placebo	Efficacy at 8 weeks	Randomized, double-blind, placebo-controlled, parallel assignment multiple-dose trial	129	SOM Biotech	France, Germany, Italy, Poland, Spain, Switzerland, UK (multi-centre)
ACTRN12621001755820	-	SLS-005 (trehalose)	Disaccharide	Early HD, ALS, SCA3	None	Efficacy at 24 weeks	Non-randomized, open-label	15-18 (4 ALS, 10 HD, 4 SCA3)	Seelos Therapeutics	Australia (two centres)
NCT05541627	-	AB-1001 (BV-101)	AAV encoding for CYP46A1, enzyme converting cholesterol to 24-OH-cholesterol	Early HD	None	Safety at week 52	Non-randomized, open-label, sequential, single ascending dose	18	AskBio/ BrainVectis	France (single centre)
NCT05107128†	DIMENSION	SAGE-718	Positive allosteric modulator of NMDA	Early and moderate HD	Placebo	Change in cognition at 84 days	Double-blind, placebo-controlled, single dose design	189	Sage Therapeutics	Australia, Canada, USA (multi-centre)
NCT05111249†	VIBRANT HD	Branaplam	Small molecule splicing modulator	Early HD	Placebo	Reduction of mHTT protein at week 17 Safety at 104 weeks	Double-blind, placebo-controlled multiple dose design	75	Novartis Pharmaceuticals	Belgium, Canada, France, Germany, Hungary, Italy, Spain, UK, USA (multi-centre)
NCT05032196†	SELECT-HD	WVE-003	Allele-selective ASO	Early HD	Placebo	Safety at 36 weeks	Randomized, double-blind, placebo-controlled, combined single ascending dose/multiple ascending dose trial	36	Wave Life Sciences Ltd	Australia, Canada, Denmark, France, Germany, Poland, Spain, UK (multi-centre)
NCT05243017	-	AMT-130	rAAV5-miHTT	Early HD	None	Safety at 6 months	Non-randomized, sequential ascending, multiple-dose trial	15	UniQure Biopharma B.V.	Germany, Poland, UK (multi-centre)
NCT04713982	-	Deutetrabenazine	VMAT2 inhibitor	HD with chorea	None	Change in speech outcome at 10 weeks	Single-arm open-label trial	30	Vanderbilt University Medical Center	USA (single centre)
NCT04826692	-	Metformin	Antihyperglycemic/ AMPK activator	Early and moderate HD	Placebo	Change in cognition at 52 weeks	Randomized, parallel assignment, double-blinded trial	60	Instituto de Investigación Sanitaria La Fe	Spain (single centre)
NCT04514367†	-	ANX005	C1q inhibitor	Early HD	None	Safety at 36 weeks	Single-dose open-label trial	28	Annexon, Inc	USA (multi-centre)
NCT04421339†	-	Melatonin	Melatonin receptor agonist	HD with sleep disturbance	Placebo	Sleep quality at 9 weeks	Randomised, cross-over, single-blinded (participant/caregiver)	20	The University of Texas Health Science Center, Houston	USA (single centre)
NCT04400331	-	Valbenazine	VMAT2 inhibitor	Early and moderate HD	None	Safety at 104 weeks	Open-label, single arm trial	150	Neurocrine Biosciences	USA, Canada (multi-centre)
NCT04301726	-	Deutetrabenazine	VMAT2 inhibitor	HD with dysphagia	Placebo	Dysphagia at 18 months	Randomized, parallel assignment, triple blinded trial	48	Fundación Huntington Puerto Rico	N/S
NCT04478734; CTIS2023-508637-14-00	HUNTIAM	Thiamine and biotin	B vitamins	HD	Moderate vs high doses of thiamine and biotin	Safety at 52 weeks	Randomized, parallel assignment, open-label trial	24	Fundación Pública Andaluza para la gestión de la Investigación en Sevilla	Spain (single centre)
NCT04201834†	-	Risperidone	Dopamine antagonist	Early and moderate HD with chorea	None	Change in motor scales at 12 weeks	Non-randomized, open-label (assessor-blind), uncontrolled trial	12	University of Rochester	USA (single centre)
NCT04071639	-	Haloperidol, risperidone, sertraline and coenzyme Q10	Multiple (dopamine antagonists, selective serotonin reuptake inhibitor, dietary supplement)	Early and moderate HD	Coenzyme Q10	Efficacy at 5 years	Randomized, open-label, controlled, parallel trial	100	Second Affiliated Hospital, School of Medicine, Zhejiang University	China (single centre)
NCT04120493	AMT-130	rAAV5-miHTT	Non-allele-selective miRNA	Early HD	Sham interv ention	Safety at 18 months	Randomized, double-blind, sham-controlled, parallel trial	26	UniQure Biopharma B.V.	USA (multi-centre)
NCT04102579†	KINECT-HD	Valbenazine	VMAT2 inhibitor	HD with chorea	Placebo	Efficacy at 12 weeks	Randomized, double-blind, placebo-controlled, parallel trial	120	Neurocrine Biosciences, Huntington Study Group	USA (multi-centre)
EUCTR2019-002178-30-DK†	-	WVE-120102	Allele-selective ASO	HD	None	Safety and tolerability at 97 weeks	Open-label extension	70	Wave Life Sciences Ltd	Australia, Canada, Denmark, France, Poland, UK (multi-centre)
NCT04000594†	GEN-PEAK	RG6042	Allele-nonselective ASO	HD	None	Pharmacodynamics and pharmacokinetics at multiple timepoints until 6 months	Non-randomized. open-label, multiple-dose, parallel trial	20	Hoffmann-La Roche	Netherlands, UK (multi-centre)
NCT03980938†	-	Neflamapimod	p38α MAPK inhibitor	Early HD	Placebo	Change in cognitive scales at 10 weeks	Randomized, double-blind, placebo-controlled, cross-over trial	16	EIP Pharma Inc, Voisin Consulting, Inc.	UK (single centre)
NCT03842969†	GEN-EXTEND	RG6042	Allele-nonselective ASO	HD	None	Safety and tolerability at up to 5 years	Open-label extension	1050	Hoffmann-La Roche	USA, Canada, Europe (multi-centre)
NCT03761849†	GENERATION-HD1	RG6042	Allele-nonselective ASO	HD	Placebo	Clinical efficacy at 101 weeks	Randomized, double-blind, placebo-controlled, parallel trial	909	Hoffmann-La Roche	USA, Canada, Europe (multi-centre)
NCT03515213†	-	Fenofibrate	PPARα agonist	HD	Placebo	Pharmacodynamics at 6 months	Randomized, double-blind, placebo-controlled, parallel trial	20	University of California, Irvine	USA (single centre)
NCT03764215	Tasigna HD	Nilotinib	Selective Bcr-Abl tyrosine kinase inhibitor	HD	None	Safety, tolerability and pharmacodynamics at 3 months	Open-label, multiple ascending dose	20	Georgetown University	USA (single centre)
NCT03225833†	PRECISION-HD1	WVE-120101	Allele-selective ASO	HD	Placebo	Safety and tolerability at 1 and 120 days	Randomized, double-blind, placebo-controlled, combined single ascending dose/multiple ascending dose trial	48	Wave Life Sciences Ltd	Australia, Canada, Denmark, France, Poland, UK (multi-centre)
NCT03225846†	PRECISION-HD2	WVE-120102	Allele-selective ASO	HD	Placebo	Safety and tolerability at 1 and 120 days	Randomized, double-blind, placebo-controlled, combined single ascending dose/multiple ascending dose trial	60	Wave Life Sciences Ltd	Australia, Canada, Denmark, France, Poland, UK (multi-centre)
NCT02453061†	TRIHEP 3	Triheptanoin	Anaplerotic therapy	HD	Safflower oil	Pharmacodynamic efficacy at 6 months	Randomized, double-blind, controlled, parallel trial	100	Institut National de la Santé Et de la Recherche Médicale, Ultragenyx Pharmaceutical Inc	France, Netherlands (multi-centre)
NCT02509793	-	Tetrabenazine	VMAT2 inhibitor	HD with impulsivity	None	Cognitive and behavioural effects at 8 weeks	Single group, open-label trial	20	University of Texas Health Science Center, and H. Lundbeck A/S	USA (single centre)
NCT02481674†	SIGNAL	VX15/2503	Anti-semaphorin 4D monoclonal antibody	Late premanifest or early HD	Placebo	Safety and tolerability at 15 and 21 months	Randomized, double-blind, placebo-controlled, parallel trial	240	Vaccinex Inc., Huntington Study Group	USA (multi-centre)
EUCTR2013-002545-10-SE	OSU6162Open1309	(-)-OSU616	Monoaminergic stabilizer	HD, PD, brain trauma, stroke, myalgic encephalomyelitis and narcolepsy	None	Safety at 3, 6 and 12 months	Single group, open-label trial	240	A. Carlsson Research AB	Sweden (multi-centre)
NCT00514774	UDCA-HD	Ursodiol	Bile acid	HD	Placebo	Safety, tolerability, and pharmacokinetics at 35 days	Randomized, double-blind, placebo-controlled, parallel trial	21	Oregon Health and Science University, Huntington Study Group, Huntington Society of Canada	N/S

AMP: adenosine monophosphate; AMPK: AMP-activated protein kinase; ASO: antisense oligonucleotide; HD: Huntington's disease; HD-ISS: Huntington's Disease Integrated Staging System; HTT: huntingtin; mRNA: messenger ribonucleic acid; NAC: N-acetylcysteine; NMDA: N-methyl-D-aspartate; N/S: not specified; RNA: ribonucleic acid; PD: Parkinson's disease; PPARα: peroxisome proliferator-activated receptor alpha; SCA1: spinocerebellar ataxia 1; SCA3: spinocerebellar ataxia 3; TD: tardive dyskinesia; TEAEs: treatment-emergent adverse events; UHDRS: Unified Huntington's Disease Rating Scale; VMAT2: vesicular monoamine transporter 2. Note: IONIS-HTT_Rx_, ISIS 443139, RG6042 and tominersen refer to the same molecule. New clinical trials added since the last Clinical Trials Update are indicated by *. Pharmacological trials terminated are indicated by †.

**Table 3.** Invasive non-pharmacological clinical trials registered at the WHO ICTRP for people with HD since the first edition of the “Clinical Trials Corner”.

Registration ID	Trial Name	Intervention	Mechanism of Action	Population	Comparison	Main Outcome	Study Design	Estimated Enrolment	Sponsor	Location(s)
NCT06444217	FibroTG-HD	Skin biopsy	Skin biopsy	Individuals with a CAG ≥36 allele (with reduced or full penetrance)	None	In vitro validation of a RNA trans-splicing gene therapy for the correction of supernumerary CAG repeats into fibroblasts from skin biopsies	Open-label, single group assignment	20	University Hospital, Angers	France (single centre)
NCT06097780	-	Nestacell	Dental pulp stem cell	Early and moderate HD	Placebo	Efficacy at 1 year	Randomized, double-blind, parallel assignment, multiple dose	120	Azidus Brasil	N/S
NCT04244513	-	GPi DBS	DBS	HD with chorea	Sham intervention	Efficacy at 3 and 6 months	Randomized, double-blind, sham-controlled, cross-over trial	40	Beijing Municipal Administration of Hospitals, Medtronic	China (multi-centre)
NCT04219241	ADORE-EXT	Cellavita	Stem cell therapy	HD	None	Efficacy and safety at 2 years	Open-label extension	35	Azidus Brasil, Cellavita Pesquisa Científica Ltda	Brazil (single centre)
ISRCTN52651778	TRIDENT	Foetal stem cell transplant	Stem cell therapy	Early HD	Usual care	Safety at 4 weeks	Randomized, open-label, controlled, parallel trial	30	Cardiff University, UK	UK (single centre)
NCT02728115	SAVE-DH	Cellavita	Stem cell therapy	HD	None	Safety at 5 years	Non-randomized, open-label, uncontrolled, parallel trial	6	Azidus Brasil	Brazil (single centre)
NCT03252535	ADORE-HD	Cellavita	Stem cell therapy	HD	Placebo	Efficacy at 120 days	Randomized, double-blind, placebo-controlled, parallel trial	35	Azidus Brasil	Brazil (single centre)
NCT03297177	-	Autologous stem/stromal cells	Autologous stem/stromal cell injection	HD, AD, PD, CBD, MS	None	Safety at 5 years	Single group, open-label trial	300	Healeon Medical Inc, Global Alliance for Regenerative Medicine, Regeneris Medical	Honduras, USA (multi-centre)
NCT02535884	HD-DBS	GP DBS	DBS	Moderate HD with chorea	Sham intervention	Efficacy at 12 months	Randomized, double-blind, sham-controlled, parallel trial	50	Heinrich-Heine University, KKS Netzwerk, Medtronic, The George Institute, EHDN, CHDI Foundation, Inc.	Austria, France Germany, Switzerland (multi-centre)
NCT01834053	BMACHC	Bone marrow derived MNC transplant	Bone marrow transplant	HD with chorea	None	Cognitive and behavioural effects at 6 months	Single group, open-label trial	50	Chaitanya Hospital, Pune	India (single centre)
NCT02252380	-	MR-guided focused ultrasound	Extracranial stereotactic radioablation	HD, ET, HT, PD, WD, dystonia, TD, or orofacial dyskinesias	None	Adverse events after the procedure	Single group, open-label trial	10	InSightec	Canada (single centre)

AD: Alzheimer's disease; CBD: corticobasal degeneration; DBS: deep brain stimulation; ET: essential tremor; GP: globus pallidus; GPi: globus pallidus internus; HD: Huntington's disease; HT: Holmes tremor; ICTRP: International Clinical Trials Research Platform; MNC: mononuclear cells; MR: magnetic resonance; MS: multiple sclerosis; N/S: not specified; RNA: ribonucleic acid; PD: Parkinson's disease; TD: tardive dyskinesia; WD: Wilson's disease; WHO: World Health Organization.

**Table 4.** Non-invasive non-pharmacological clinical trials registered at the WHO ICTRP for people with HD since the first edition of the “Clinical Trials Corner”.

Registration ID	Trial Name	Intervention	Mechanism of Action	Population	Comparison	Main Outcome	Study Design	Estimated Enrolment	Sponsor	Location(s)
NCT06774443*	HT-HD	Neuropsychological assessment (Hinting Task)	Assessment of social cognition deficits, specifically Theory of Mind	HD	Healthy controls	Sensitivity of the Hinting Task in detecting social cognition impairments in HD	Observational (case-control, cross-sectional)	52	University Medical Center Groningen	Netherlands (single centre)
NCT06693466*	HD-P1	Experimental pain assessment (pain facilitation, facial expression, and conditioned pain modulation)	Assessment of pain processing mechanisms and sensory modulation in HD	HD	None	Feasibility of experimental pain assessment protocols in HD	Observational (cohort, cross-sectional)	20	Leiden University Medical Center	Netherlands (single centre)
NCT06634628*	iMagemHTT-009	PET imaging with novel radioligand [11C]CHDI-00491009	Radioligand binding to aggregated mHTT for quantification	HD	None	VT of [11C]CHDI-00491009 measured via PET imaging	Non-randomized, sequential assignment	27	CHDI Foundation, Inc.	Belgium (single centre)
NCT06626308*	BIO-MH	Brain fMRI and gait assessment using 3D virtual reality	fMRI to assess early subcortical biomarkers and virtual reality-based gait analysis for detecting preclinical motor abnormalities	Premanifest HD	Healthy controls	BOLD signal changes in subcortical regions and alterations in gait parameters	Observational (case-control)	20	University Hospital, Grenoble	France (single centre)
NCT06626412*	-	PET of SV2A using the radioligand 18F-SynVesT-1	Radioligand binding to SV2A for quantification of synaptic density	Late premanifest HD	Volumetric MRI	Baseline differences and longitudinal changes in synaptic density and brain volume	Non-randomized, single-group assignment	35	Universitaire Ziekenhuizen KU Leuven	Belgium (single centre)
NCT06585332*	-	Hand grip strength as a screening tool for respiratory dysfunction	Assessment of neuromuscular function related to respiratory effort	HD	None	Maximal hand grip strength, voluntary peak cough flow, maximal expiratory pressure, maximal inspiratory pressure	Observational (case only)	70	General University Hospital	Prague (single centre)
NCT06546488*	CAT-HD	Behavioral assessments	Standardised assessment batteries using the DSCT and SAGE	HD	SDMT, SWR	SDMT compared to DSCT; SAGE score compared to SDMT and SWR scores	Observational (case only)	76	Ohio State University	USA (single centre)
NCT06490367	TREHD	TRE diet	TRE diet, specifically maintaining a 6-8-h eating window every day for 12 weeks	Premanifest, prodromal and early HD	None	Change from baseline in the daily eating period at week 13	Prospective interventional, open-label, single-arm trial	25	Oregon Health and Science University	USA (single centre)
NCT06414967	MUSIC-HD	Music therapy	Music therapy using a digital music therapy tool combined with conventional management	Early HD	None	Change in irritability after 3 months	Single group, open-label	15	Poitiers University Hospital	France (single centre)
RBR-75ys4s9	-	Dance therapy	Dance sessions offered by physiotherapists using auditory and visual stimuli	HD, PD, dystonia	Standard medical treatment	Quality of life improvement	Two-arm randomized controlled trial with a blinded examiner	100	Universidade Federal de Juiz de Fora	Brazil (single centre)
ChiCTR2300069844	-	Repetitive transcranial magnetic stimulation	Transcranial magnetic stimulation	HD	None	EEG	Non-randomized, open-label, single group trial	20	Shenzhen People's Hospital	China (single centre)
ISRCTN47330596	-	Psychological intervention	Guided self help	Premanifest and manifest HD	Usual treatment	Feasibility at 3 and 6 months	Interventional randomized controlled trial	30	Leicestershire Partnership NHS Trust, UK	UK (single centre)
RBR-463yhb3	-	Multimodal physiotherapy	Balance intervention with rhythmic cues	HD	Educational program	Balance	Randomized, double-blinded, parallel assignment trial	36	São Paulo University, Brazil	Brazil (single centre)
ACTRN12622000908730	-	Online platform	Computerized cognitive training	Premanifest and early HD	Lifestyle education	Change in cognition at 12 weeks	Randomized, blinded (investigator, statistician) parallel assignment trial	50	Monash University, Australia	Australia (two centres)
ISRCTN11906973	HD-DRUM	Training app	Drumming	Premanifest, early and moderate HD	Standard medical care	Feasibility	Randomized, parallel assignment trial	50	Cardiff University, UK	UK (single centre)
NCT05326451	-	Transcranial direct current stimulation	Transcranial electrical stimulation	Early and moderate HD	None	Treatment completion, acceptability and safety	Non-randomized, open-label, single group trial	10	The University of Texas Health Science Center, Houston, USA	USA (single centre)
ACTRN12622000345785	-	Multidisciplinary therapy coaching program	Education	Premanifest and early HD	Lifestyle guidance	Barriers and motivators to engagement in telehealth interventions and digital health literacy	Randomized, single blind, parallel assignment trial	84	Perpetual limited	Australia (two centres)
NCT04917133	HUNT'ACTIV	Adapted physical workshops plus classic 4-week rehabilitation program	Physical activity, cycling, horse riding, situation tests, cultural outings	Mid-stage HD	Classic 4-week rehabilitation program	Motor function at 1 month	Randomized, parallel assignment trial	32	Assistance Publique - Hôpitaux de Paris	France (single centre)
NCT04429230	-	Transcranial pulsed current stimulation	Transcranial electrical stimulation	HD	Sham intervention	Feasibility at one year	Randomized, crossover double-blinded trial	15	Western University, Canada	N/S
ACTRN12620000281998	-	Ketogenic diet	Ketogenic diet	HD	None	Change in cognition and motor scores at 12 weeks	Non-randomized, open-label, single group trial	10	Waikato Hospital	New Zealand (-)
ACTRN12619000870156	-	Transcranial alternating current stimulation	Transcranial magnetic stimulation	Premanifest and early HD	Sham intervention	Biomarkers	Randomized, open-label, cross-over trials	60	Monash University, Epworth Centre for Innovation in Mental Health	Australia (single centre)
ACTRN12618001717246	-	Multidisciplinary therapy program	Exercise, cognitive training, lifestyle guidance and social activities	Premanifest HD	Standard of care	Feasibility and safety	Clustered, non-randomized, open-label, parallel trial	40	Edith Cowan University, Deakin University and Lotterywest	Australia (two centres)
NCT03417583	-	Neuropsychiatric treatment protocol	Multidisciplinary intervention	HD with neuropsy chiatric symptoms	Standard of care	Change in quality of life at 18 months	Non-randomized, assessor-blinded, parallel trial	100	Vanderbilt University Medical Center and Teva Pharmaceu ticals USA	USA (single centre)
CTRI/2018/01/011359	-	Repetitive transcranial magnetic stimulation	Transcranial magnetic stimulation	Early to moderate HD and PD	Sham stimulation	Efficacy at 5 days	Randomized, single-blind, placebo-controlled, parallel trial	40	Vinay Goyal	India (single centre)
NCT03344601	PACE-HD	Supported structured aerobic exercise training program	Physiotherapy	HD	Activity as usual	Data completeness, recruitment, retention, safety, adherence, fidelity and acceptability at 12 months	Nested open-label, randomized controlled parallel trial	120	Cardiff University and CHDI Foundation, Inc	Germany, Spain, USA (multi-centre)
ACTRN12617001269325	-	Swallowing skill training	Speech and language therapy	HD and ALS	None	Swallowing function and quality of life at 2 weeks	Single group, open-label trial	54	University of Canterbury	New Zealand (single centre)

3D: three-dimensional; AD: Alzheimer's disease; ALS: amyotrophic lateral sclerosis; BOLD: blood oxygen level–dependent; DSCT: Digit Symbol Coding Test; EEG: electroencephalography; ET: essential tremor; fMRI: functional magnetic resonance imaging; HD: Huntington's disease; HT: Holmes tremor; ICTRP: International Clinical Trials Research Platform; mHTT: mutant huntingtin; MRI: magnetic resonance imaging; MS: multiple sclerosis; N/S: not specified; PD: Parkinson's disease; PET: positron emission tomography; SAGE: Self-Administered Gerocognitive Examination; SDMT: Symbol Digit Modalities Test; SV2A: synaptic vesicle glycoprotein 2A; SWR: Stroop Word Reading; TD: tardive dyskinesia; TRE: time-restricted eating; VT: volume of distribution; WHO: World Health Organization. New clinical trials since the last Clinical Trials Update are indicated by *.

If you would like to draw attention to specific trials, please feel free to email us at: mena.farag@ucl.ac.uk and e.wild@ucl.ac.uk.

## Ongoing clinical trials

A list of all registered clinical trials is given in Tables 2, 3 and 4.

## ALN-HTT02 (NCT06585449)^12^

**Study Title:** A Study to Evaluate ALN-HTT02 in Adult Patients With Huntington's Disease.

**Intervention:** A single-dose intrathecal administration of ALN-HTT02, a synthetic double-stranded RNA interference therapy conjugated with C16 for enhanced CNS delivery, designed to target and specifically bind to exon 1 of *HTT* mRNA.

**Description:** The ALN-HTT02 clinical trial, sponsored by Alnylam Pharmaceuticals, is a phase Ib, first-in-human study designed to evaluate the safety, tolerability, pharmacokinetics and pharmacodynamics of a single intrathecal ascending dose of ALN-HTT02 in people with HD. This randomised, double-blind, placebo-controlled clinical trial follows a parallel assignment interventional model and aims to recruit 54 study participants aged 25 to 70 years with HD Integrated Staging System (HD-ISS)^16^ stage 2 or early stage 3 HD.

ALN-HTT02 is a synthetic double-stranded RNA interference therapy designed to target exon 1 of *HTT* mRNA, leading to a non-selective reduction of both mutant (mHTT) and wild-type HTT proteins including, in principle, the exon 1 missplice variant sometimes known as HTT1a. Conjugated with C16 to enhance CNS delivery, ALN-HTT02 is administered intrathecally and expected to have a half-life consistent with twice-yearly or even less frequent dosing. Participants who receive placebo during the double-blind phase will have the option to receive one dose of ALN-HTT02 in an open-label extension.

The primary outcome measure is the frequency of adverse events (AEs) over 12 months in both the double-blind and open-label study phases. Secondary outcome measures include changes in cerebrospinal fluid (CSF) mHTT levels, as well as CSF, plasma and urine concentrations of ALN-HTT02, all assessed over 12 months in both study phases.

Recruitment for the clinical trial commenced on 14^th^ October 2024, with an estimated study completion date of 5^th^ July 2028. Currently, the study is actively being conducted across two locations, in the UK and Canada, and is expected to expand globally.

**Sponsor/Funders:** Alynlam Pharmaceuticals

**Comments:** The RNA interference mechanism leverages an endogenous cellular process to reduce the expression of disease-associated target proteins. ALN-HTT02 specifically targets a conserved mRNA sequence within exon 1 of *HTT*, leading to the lowering of all HTT protein species, including all canonical mHTT and wild-type HTT proteins and all known splice variants.

The first program to utilise Alnylam's proprietary C16-siRNA technology was ALN-APP (NCT05231785),^17^ in collaboration with Regeneron, designed to target amyloid precursor protein (APP) for the potential treatment of Alzheimer's disease (AD) and cerebral amyloid angiopathy (CAA). The phase I clinical trial employed a double-blind, placebo-controlled, single ascending dose approach with the aim to assess the safety, tolerability, pharmacokinetic and pharmacodynamic effects of ALN-APP in patients with early-onset AD;^12^ the ALN-HTT02 trial has a similar design. Interim results for ALN-APP, announced by Alnylam Pharmaceuticals in a press release on 25^th^ October 2023,^18^ reported that single doses demonstrated sustained pharmacodynamic activity for up to 10 months post-administration. In addition, the treatment led to marked reductions in Aβ42 and Aβ40, key proteins implicated in progression of AD and CAA. From a safety perspective, among the 20 patients randomised to receive ALN-APP or placebo, one subject experienced two mild AEs deemed related to both the study drug and procedure, but no serious AEs or deaths were reported.^19^ These interim results for ALN-APP represent the first-in-human translation of an RNA interference therapeutic for neurodegenerative diseases, with the ongoing ALN-HTT02 clinical trial in HD expected to improve understanding of the approach and potential therapeutic benefits of this strategy.

## Completed clinical trials

### SAGE-718 (NCT05107128; NCT05655520)^13,14^

**Study Title:** A Study to Evaluate the Effect of SAGE-718 on Cognitive Function in Participants With Huntington's Disease; and A Study to Evaluate the Safety and Tolerability of SAGE-718 in Participants With Huntington's Disease.

**Intervention:** Daily oral administration of SAGE-718 (also known as dalzanemdor), a positive allosteric modulator of *N*-Methyl-d-aspartate receptor (NMDAR), designed to enhance NMDAR function.^20^

**Description:** The DIMENSION study, sponsored by Sage Therapeutics, was a phase II clinical trial designed to evaluate the effect of SAGE-718 cognitive function in people with HD. This randomised, double-blind, placebo-controlled clinical trial enrolled 189 participants with HD, aged 25 to 65 years, who met the inclusion criteria of genetically confirmed HD (CAG repeat length ≥36). Eligible participants had a Unified Huntington's Disease Rating Scale (UHDRS) Total Functional Capacity (TFC) score between 6 and 13, indicating no more than moderate functional impairment, a Montreal Cognitive Assessment (MoCA) score of 15 to 25, consistent with cognitive impairment, and were ambulatory. Participants were randomised to receive either oral SAGE-718 or placebo once daily for 84 days.

The primary outcome measure was the change from baseline in the Symbol Digit Modalities Test (SDMT) score, with higher scores indicating greater cognitive processing speed. Secondary outcomes included changes from baseline in clinical, cognitive and functional assessments. Functional independence was assessed using the UHDRS Independence Scale (IS), where higher scores indicated greater function. Cognitive performance was assessed through the Trail Making Test Part B of the MoCA and the One Touch Stockings of Cambridge (OTS) task, with longer completion times reflecting greater impairment. Motor timing accuracy was measured using the Paced Tapping Test (PTAP), where higher scores indicated improved precision. Functional capacity in daily life was assessed using the HD Everyday Functioning (Hi-DEF) Home Subdomain score, with lower scores reflecting better functional abilities. The Clinical Global Impression – Severity (CGI-S) Cognitive Status Subdomain score was used to assess cognitive symptom severity, with higher scores indicating greater impairment. Safety and tolerability were also assessed as a secondary outcome measure by monitoring the incidence of treatment-emergent AEs (TEAEs).

Recruitment for the clinical trial began on 10^th^ February 2022 and was completed on 3^rd^ October 2024. The study was conducted across 45 sites.

**Sponsor/Funders:** Sage Therapeutics

**Comments:** On 20^th^ November 2024, Sage Therapeutics announced the results of the phase II DIMENSION study.^21^ In summary, dalzanemdor did not demonstrate a statistically significant difference compared to placebo on the primary endpoint, the change from baseline in the SDMT at 12 weeks. Additionally, analyses of secondary endpoints showed no statistically significant or clinically meaningful differences between dalzanemdor and placebo. Dalzanemdor was generally well tolerated, with no new safety signals reported. Based on these findings, Sage Therapeutics announced that further development of dalzanemdor would not proceed. As a result, the PURVIEW study, an open-label rollover safety study of dalzanemdor in participants with Huntington's disease,^14^ was discontinued and terminated on 10^th^ January 2025. This disappointing outcome means the substantial need for treatments for impaired cognition in people with HD remains unmet.

## Breaking news

In this section we provide brief updates about ongoing or recently terminated clinical trials.

The **uniQure AMT-130** program (NCT04120493; NCT05243017) is investigating the effects of AMT-130, a gene therapy that uses a viral vector (AAV5-miHTT) to deliver a microRNA targeting the *HTT* gene.^22,23^ This treatment is administered intracranially, with its effects anticipated to persist for several years. In the September 2024 issue of the Huntington's Disease Clinical Trials Update,^11^ we discussed 24-month interim results from the program.

On 10^th^ December 2024, uniQure provided an optimistic update on its discussions with the U.S. Food and Drug Administration (FDA) regarding key aspects of the accelerated approval pathway for AMT-130 in HD.^15^ During a Regenerative Medicine Advanced Therapy (RMAT) Type B meeting held in late November 2024, the FDA agreed that data from the ongoing phase I/II studies, compared against an external natural history control, could serve as the primary evidence for a Biologics License Application (BLA) submission under the Accelerated Approval pathway. The FDA also endorsed the use of composite UHDRS (cUHDRS)^24^ as an intermediate clinical endpoint, which is a composite measure integrates scores from multiple assessments, including the UHDRS TFC, Total Motor Score (TMS), SDMT and Stroop Word Reading (SWR). The FDA also acknowledged that reductions in CSF neurofilament light chain (NfL) could serve as supportive evidence of therapeutic benefit, given supportive evidence with reductions in CSF NfL levels observed in treated participants at 24 months relative to baseline.^25^

While it cannot be assumed that statements made in a RMAT meeting with a single sponsor will automatically apply to other programs, it is highly encouraging to learn of regulatory acceptance in principle for both cUHDRS and NfL. FDA previously rejected the use of cUHDRS as a primary efficacy endpoint, but its use in place of TFC is expected to reduce clinical trial participant number requirements by over 50%. Similarly, NfL is widely seen as a reliable indicator of neuronal damage and rescue in other CNS indications, and has been used to inform regulatory decisions (e.g., for tofersen in amyotrophic lateral sclerosis); it could be highly valuable for therapeutic development for it to receive similar consideration in HD.
